# Surgical Treatment of Vertical Shear Pelvic Fracture Associated with a Lumbosacral Plexus Injury through the Lateral‐Rectus Approach: Surgical Techniques and Preliminary Outcomes

**DOI:** 10.1111/os.13359

**Published:** 2022-06-30

**Authors:** Xiaorui Zhan, Kangshuai Xu, Qiubao Zheng, Sheqiang Chen, Jiacheng Li, Hai Huang, Yuhui Chen, Cheng Yang, Shicai Fan

**Affiliations:** ^1^ Department of Traumatic Surgery Center for Orthopaedic Surgery, The Third Affiliated Hospital of Southern Medical University Guangzhou China; ^2^ Department of Orthopedic Huizhou First Hospital Huizhou China; ^3^ Department of Orthopedic Panyu District Central Hospital Guangzhou China

**Keywords:** Lateral rectus approach, Lumbosacral plexus injury, Pelvic fracture, Vertical shear

## Abstract

**Objective:**

To examine the surgical techniques and preliminary outcomes of the lateral rectus approach (LRA) for treating vertical shear (*VS*) pelvic fracture associated with lumbosacral plexus (LSP) injury.

**Methods:**

This study was a retrospective trial. From August 2010 to October 2017, 29 patients with *VS* pelvic fractures involving LSP injury who were treated with the LRA were included in this study. The patients were 18–61 years old, with a mean age of 36.2 years. All patients underwent neurolysis, open reduction, and internal fixation (ORIF) through the LRA. The fracture reduction was evaluated using the Matta criteria, and the neural recovery was evaluated by muscle strength grading proposed by the British Medical Research Council (BMRC).

**Results:**

All 29 patients underwent the surgery successfully. The mean operating time was 155.2 ± 32.1 min (range: 105–220 min). The mean operative blood loss was 1021.4 ± 363.4 mL (range: 400–2000 mL). All patients were followed‐up for at least 24 months (mean, 32.8 ± 13.5 months; range: 24–96 months). According to the Matta criteria, there were 17 excellent cases, nine good cases, and three fair cases in 29 patients. The ratio of excellent‐to‐good cases was 89.66%. According to the criteria of the Nerve Injuries Committee of the British Medical Research Council (BMRC), the recovery of nerve and muscle strength achieved to M5 (full recovery of neurological symptoms) was 14 cases, M4 (fine recovery of neurological symptoms), seven cases; M1, M2, and M3 (partial recovery of neurological symptoms), five cases, and M0 (no recovery of neurological symptoms), three cases.

**Conclusions:**

LRA is a safe and feasible surgical approach for treating *VS* pelvic fractures with LSP injury, which can be used to perform nerve exploration and release from the front, reduce the fracture, and fix it with the anterior iliac plates and/or sacroiliac screws.

## Graphical abstract

The surgical field of LRA.

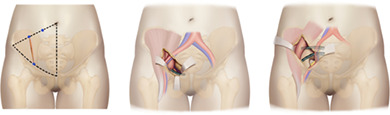

## Introduction

Vertical shear (*VS*) pelvic fracture is frequently caused by high energy shear force and is characterized by severe instability of the pelvic ring vertically and rotationally. In this type of fracture, the rate of lumbosacral plexus (LSP) injury is approximately 50% due to the lumbosacral trunk (LST) being situated medially to the surface of sacroiliac joint and track along the pelvic margin under a high level of tension.^1^, ^2^ In addition, the vast distribution of vessels results in a high risk of surgical decompression and neurolysis of LSP injury in the *VS* pelvic fracture.

With improvements in the early rescue of pelvic fractures and the pre‐hospital survival rate in patients with severe pelvic injuries, the treatment of pelvic fractures combined with LSP injury has received increasing attention. However, treating pelvic fractures combined with LSP injury is still controversial.^3^, ^4^ First, is early surgical decompression and neurolysis of LSP necessary for the patient? Second, is this the appropriate selection of surgical approach to treat such injuries? Currently, most surgeons expose and correct pelvic fractures through the posterior approach, to maintain stability with lumbo‐sacral or triangular fixation. However, posterior fixation cannot easily correct the rotational shift of the pelvis; it also affects the structural integrity and mobility of the lumbar spine, and it cannot effectively release the nerve compression caused by anterior compression. Non‐surgical treatment or decompression through the posterior iliac nerve hole is currently used for combined LSP injury. Some patients receiving non‐surgical treatment miss the opportunity for early treatment, and posterior decompression cannot enable direct visualization of the anterior iliac region and allow the anteriorly displaced bone mass to be effectively removed. Thus, these two treatments tend not to achieve ideal surgical results.

The most widely used anterior approaches for pelvic fractures include the iliac fossa approach, the ilioinguinal approach, and the Stoppa approach. However, none of them can achieve sufficient nerve exposure. The lateral rectus approach (LRA) is a valuable alternative to the ilioinguinal and modified Stoppa approaches. In addition, it is a novel anterior approach that can properly expose the medial pelvic from the sacroiliac joint to the symphysis pubis, allow direct release of the lumbosacral plexus nerve to be compressed and stretched, and lead to satisfactory fracture reduction. And it may be a better choice for the patients suffering from the *VS* pelvic fracture associated with LSP injury.

The purposes of this study were as follows: (i) evaluate the surgical techniques and preliminary outcomes of the LRA in treating *VS* pelvic fracture associated with LSP injury; and (ii) evaluate the safety and feasibility of the LRA for treating *VS* pelvic fractures with LSP injury.

## Materials and Methods

### Patients

This study was implemented with the approval of the Ethics Committee of the Third Affiliated Hospital of Southern Medical University (approval No. 201508006). All procedures involving human participants were in accordance with the ethical standards of the Local Institutional and National Research Committee and with the 1964 Helsinki Declaration and its later amendments. Informed consent was obtained from all participants included in the study.

All patients underwent radiographic examination including anterior–posterior (AP), inlet, and outlet views X‐rays, computed tomography (CT) scan (slice thickness of 1 mm) and magnetic resonance imaging (MRI) presurgery. The fractures were classified according to the Young–Burgess classification^5^, ^6^ and Tile classification.^2^ The cases were all *VS* pelvic fractures according to the Young–Burgess classification. According to Tile classification, the cases included Tile C1.3 (24 pelvises), Tile C2 (four pelvises) and Tile C3 (one pelvis). All patients were combined with ipsilateral LSP injury, including eight cases of complete injury and 21 cases of partial injury, according to nerve injury classification. Among them, one case was combined with ipsilateral acetabular fractures, three with ipsilateral femoral neck fractures, eight with other fractures, and 10 with other organ injuries. Immediately after admission, a pelvic external fixator (21/29) was fixed and femoral condyle traction was performed on the affected side for fracture reduction and movement restriction (25/29). These data, as well as mechanisms of injury and the time from injury to surgery, are shown in Table 1.

**TABLE 1:** Patient demographic and injury data

Parameter	Value	Percent (%)
Age (years)	36.2 (18–61)	
Sex		
Male	21	72.4
Female	8	27.6
Mechanism of injury		
Traffic accident	12	41.4
Fall from height (greater than standing)	10	34.5
Fall (from standing height)	1	3.4
Injury by heavy objects	6	20.7
Time from injury to surgery(days)	11.4 ± 3.9 (range: 7–20)	
Fracture classification		
*VS*	29	100
Tile C1.3	24	82.8
Tile C2	4	13.8
Tile C2	1	3.4
Multiple injuries		
Ipsilateral LSP injury	29	100
Complete injury	8	27.6
Partial injury	21	72.4
Ipsilateral acetabular fractures	1	3.4
Ipsilateral femoral neck fractures	3	10.3
Other fractures	8	27.6
Other organ injuries	10	34.5
Preoperative management		
External fixator	21	72.4
Femoral condyle traction	25	86.2

### Inclusion and Exclusion Criteria

Inclusion criteria: (i) acute fracture (<21 days) and *VS* injury of the pelvic ring with obvious vertical shift; (ii) injuries combined with LSP injury (physical examination, CT, and MRI all suggest LSP injury); and (iii) surgical treatment with the LRA.

Exclusion criteria: (i) the sacrum fracture involved the posterior sacrum foramen with imaging showing that a fracture block was embedded in the sacrum foramen, which required posterior decompression and neurolysis; (ii) the ipsilateral abdomen had undergone surgery; and (iii) the patient was treated by an anterior–posterior combined approach or conservative treatment.

### Perioperative Management

Related routine and preoperative examinations were performed immediately after the patients' confirmation. Standard pelvic CT scan (Toshiba 64 lines, 1 mm) were performed on all patients and then the original data were inputted (Dicom standard) into Mimics software (Materialise, 15.0). After 3‐D reconstruction through Mimics software, 1:1 3‐D pelvic models were manufactured by a 3‐D printer (Stratasys Dimension 1200es), which can clearly and certainly indicate the fracture types, positions of fractures, and displacements from different angles. For preoperative planning, precondylar plate fixation was prepared if the residual space of the sacral wing was enough to place two screws. Surgeons can perform the simulated operation *in vitro* to correct the fracture on a pelvic model and preshape the plate.

Surgery was performed with conventional anticoagulation treatment after the patient's systemic condition was stabilized. Rivaroxaban (10 mg, qd) was given to prevent deep vein thrombosis. To ensure against it, the Doppler vascular examination was performed again 1 day presurgery, along with a cleansing enema. Withholding oral ingestion of food and fluids for 8 h was performed the night before the operation. A broad spectrum antibiotic was given 30 min before the operation. If the operative time was more than 3 h or intraoperative blood loss more than 1000 mL, the antibiotic would be given again.

### Surgical technique

After tracheal intubation general anesthesia, patients were placed in the supine position on a radiolucent operating table. All fractures were exposed using LRA. The surgery was performed by the same physician.

#### Exposure, Reduction of the Fracture and Release of the Nerve

**Fig. 1:**
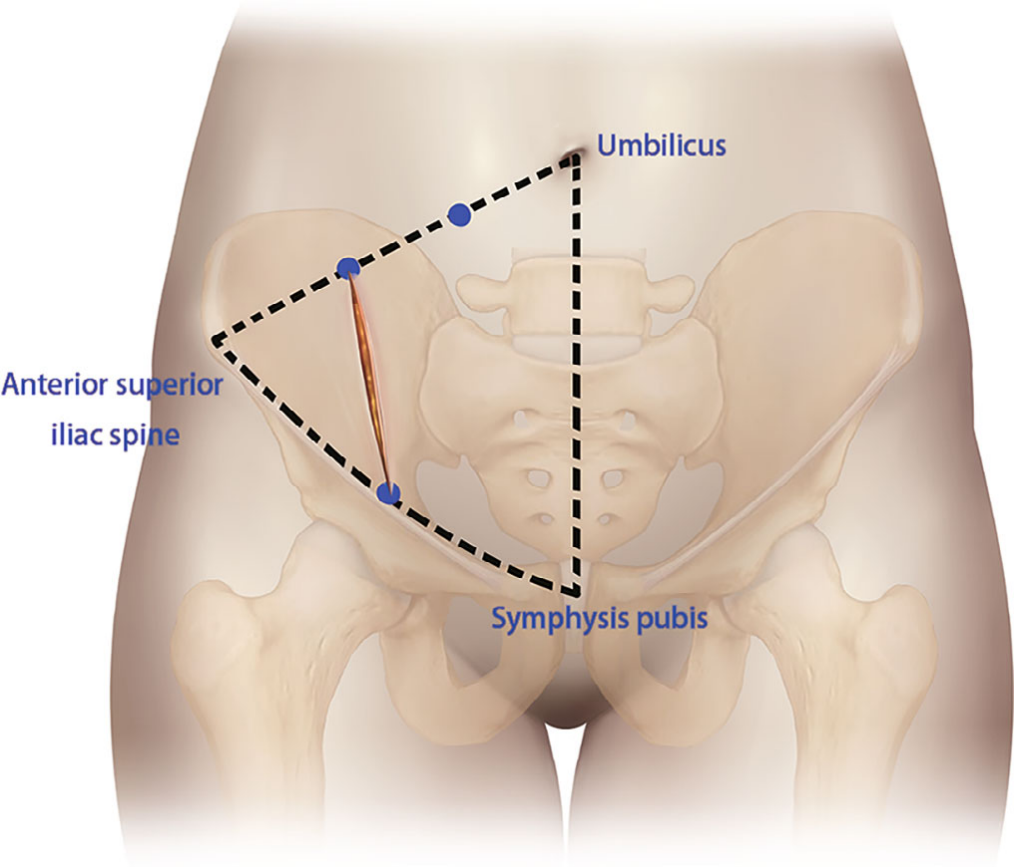
Surgical skin incision used in the lateral‐rectus approach

**Fig. 2:**
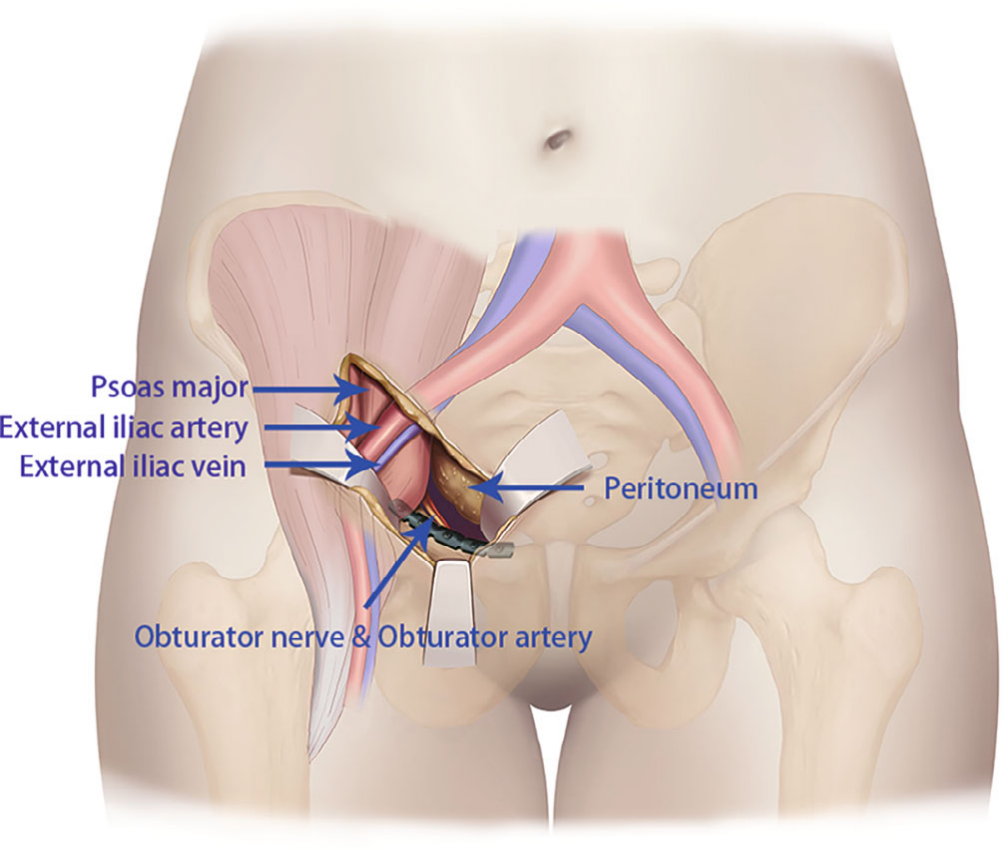
Medial window of the lateral‐rectus approach

**Fig. 3:**
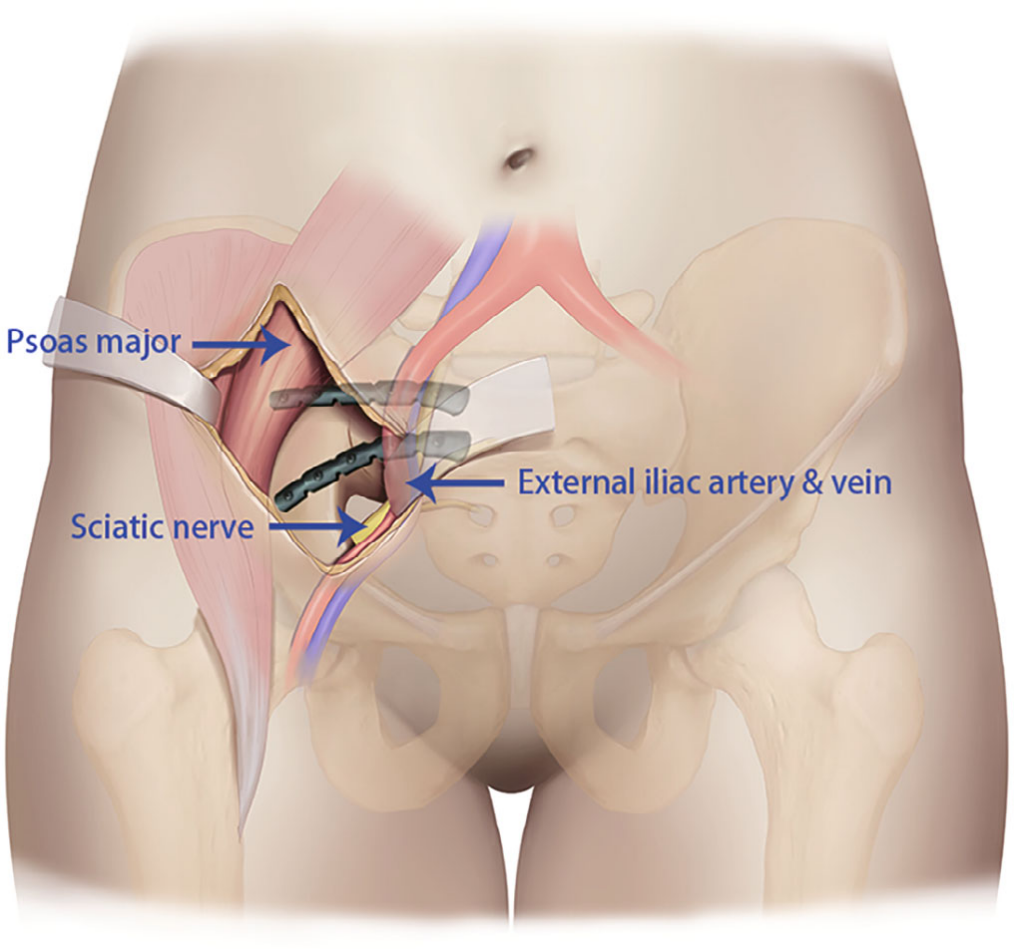
Middle window of the lateral‐rectus approach

Incision of the lateral‐rectus approach starts at the point located two‐thirds of the distance from the umbilicus to the anterior superior iliac spine and ends at the midpoint of the inguinal ligament. The incision is along the lateral border of the rectus abdominis muscle and is from 5 to 10 cm long (Fig. 1). The incision can be extended upward if there is not enough fracture exposure. The extraperitoneal space is entered after subcutaneous dissection and oblique split of the obliquus externus abdominis, obliquus internus abdominis, transverse abdominis, and ends at the medial margin of the superficial inguinal ring.^7^ The medial window, which exposed the area from the iliopectineal eminence to the symphysis pubis, was situated between the external iliac vessels and the obturator neurovascular structures. The anterior pelvic ring was exposed through the window and the soft tissue surrounding the anterior ring fracture was released (Fig. 2). The middle window was located between the iliopsoas muscle and external iliac vessels (Fig. 3). This window exposed the sacrum, sacroiliac joint, medial aspect of the posterior column, and quadrilateral plate *via* lateral retraction of the iliopsoas muscle and medial retraction of the external iliac vessels, lumbar plexus, and obturator nerve and vessels. The tissue around the sacroiliac joint was exposed through this window, and it is peeled along the sacroiliac joint under the periosteum to the medial patella auricular surface. While probing and releasing the LSP, bleeding from the anterior iliac venous plexus can be stopped by compression or bipolar electrocoagulation. After the fracture ends were exposed, the soft tissue at the fracture ends was cleaned up. The LSP compressed in the fracture ends was lifted and protected. The fracture was corrected by prying the fracture ends, linkage reduction of anterior and posterior rings, and assisted lower limb traction (a pelvis‐free reduction frame was used for assistance in fractures more than 2 weeks old).

#### Fixed Fractures

Fixation of the anterior iliac plate or sacroiliac screw was used, as appropriate. The former was preferred for the sacral Denis I fracture, while the S1 (and S2) screws fixation were used for the sacral Denis II and III fractures under direct vision and X‐ray. Decompression and neurolysis of the lumbosacral neural trunk were checked after fracture fixation. If compression was found in the anterior S1 foramen before surgery, decompression and neurolysis of the S1 foramen were performed at the same time. Then the plates or front pillar screws were used to correct the anterior ring. Acetabular fracture reduction and fixation were performed using the same incision, combined with the ipsilateral acetabular fracture. Fracture reduction and fixation could be performed with assistance from contralateral LRA combined with the bilateral pelvic fracture.

### Postoperative Management

After the operation, the lower limbs were checked to ensure equal length and no rotational malformation. Both lower limbs were lifted up with hip flexion to reduce femoral vascular tension. An indwelling drainage tube was used after the operation and would be removed when the drainage volume became less than 50 mL daily. Liquid diet was allowed after anal passage of gas, and laxative was given to prevent constipation. Rivaroxaban is used routinely 6 h postoperatively to prevent deep venous thrombosis of the lower limbs. Depending on the improvement in nerve function, physical therapy such as acupuncture or medium‐frequency electrical stimulation was performed 1 day postoperatively to promote recovery of nerve function. Pelvic X‐ray (anterior–posterior, inlet, and outlet views) and CT scan were performed to evaluate fracture reduction. Nerve function was measured to check for iatrogenic nerve injury.

### Efficacy Evaluation

#### Matta Criteria

Postoperative pelvic fracture reduction quality was assessed according to the Matta criteria. Pelvic X‐ray and CT scan were performed postoperatively to evaluate fracture healing. The quality of fracture reduction was measured using the Matta criteria according to the maximal shift measured on the three standard views of the pelvis.^8^ The results were divided into four levels: excellent (4 mm or less), good (5 to 10 mm), fair (10 to 20 mm), and poor (more than 20 mm).

#### Muscle strength grading

Postoperative muscle strength was evaluated using the muscle strength grading proposed by the Nerve Injuries Committee of the British Medical Research Council (BMRC).^9^ The grading system was divided into six levels as follows. M0 (none) represents no evidence of contractility; M1 (trace) represents evidence of slight contractility no joint motion, or return of perceptible contraction of the proximal muscles; M2 (poor) represents complete range of motion (ROM) with gravity eliminated, proximal muscle contraction against gravity, and perceptible distal muscle contraction. M3 (fair) represents complete ROM against gravity and return of function in proximal and distal muscles to such a degree that all important muscles are sufficiently powerful against gravity. M4 (good) represents complete ROM against gravity with some resistance, all muscles acting against strong resistance, and some independent movements are possible, but with some intrinsic weakness. M5 (normal) represents complete range of motion against gravity with full resistance, and full recovery in all muscles(Table 2).

**TABLE 2:** Muscle strength grading^a^

Level	Improved degree
M0	None. No evidence of contractility.
M1	Trace. Evidence of slight contractility. No joint motion. Return of perceptible contraction of the proximal muscles.
M1+	Proximal muscles contract against gravity but distal muscles are paralyzed.
M2	Poor. Complete range of motion with gravity eliminated. Same as M1+ with perceptible distal muscles contraction.
M2+	Proximal and distal muscles are all active against gravity.
M3	Fair. Complete range of motion against gravity. Return of function in proximal and distal muscles to such a degree that all important muscles to such a degree that all important muscles are sufficiently powerful against gravity.
M4	Good. Complete range of motion against gravity with some resistance. All muscles act against strong resistance and some independent movements are possible; some intrinsic weakness.
M5	Normal. Complete range of motion against gravity with full resistance. Full recovery in all muscles.

^a^ British Medical Research Council (BMRC) classification.

### Statistical analysis

Data are reported as numbers and percentages for categorical variables, and continuous variables are presented as means and standard deviations (SD). All statistical analyses were conducted with SPSS 23.0 (IBM Corp, Chicago, Ill, USA).

## Results

### General Results

Twenty‐nine patients were treated successfully with open reduction and internal fixation (ORIF) by the lateral‐rectus approach. The mean time from fracture to surgery was 11.4 ± 3.9 days (range: 7–20 days). The mean operating time was 155.2 ± 32.1 min (range:105–220 min). The mean operative blood loss was 1021.4 ± 363.4 mL (range: 400–2000 mL). All patients were followed‐up for at least 24 months (mean, 32.8 ± 13.5 months; range: 24–96 months).

### Intraoperative Results

During the operation, we found sacral plexus compression in nine cases, nerve stretch injury caused by fracture displacement in 16 cases, and radicular avulsion in four cases. Neurolysis and decompression were completed for nerve compression and stretch injury, while avulsion of the nerve root was not repaired. There was no iatrogenic nerve injury.

### Fracture Fixation and Healing Status

Internal fixation of pelvic ring fractures: anterior ring plate + posterior ring single plate fixation (six cases), anterior ring plate + posterior ring double plate fixation (three cases), anterior ring plate + posterior ring sacroiliac screw fixation (15 cases). In the bilateral posterior ring fracture (five cases), sacroiliac screw + plate fixation (four cases), plate + plate fixation (one case).

### Matta Criteria

According to Matta imaging grading, there were 17 excellent cases, nine good cases and three fair cases in 29 patients. The ratio of excellent and good cases was 89.66%. During the follow‐up, fracture reduction loss was found in three cases, and nonunion combined with reduction loss was found in one case. Posterior lumbosacral fixation and fusion with bone grafting were performed 1 year postoperatively. Bony union was formed in all the other cases with a mean healing time of 10.2 ± 1.34 weeks (range: 8–14 weeks).

### Recovery of Neural Function

At 2‐year follow‐up, 14 patients had recovered completely (muscle strength restored to M5), seven patients had recovered well (M4), three patients had recovered partially (M1–M3), and one patient had not recovered (muscle strength restored to M0) in the 25 cases of compression or stretch nerve injury. In the four cases of radicular avulsion, two patients had recovered partially (which may be related to neural cross‐domination), while two patients had not recovered. Results of the last follow‐up: 14 patients had recovered completely, seven patients had recovered well; five patients had recovered partially, and three patients had not recovered.

### Complications

Peritoneal injury occurred in three cases during the operation and there were no related complications after suturing. Incision fat liquefaction occurred in two cases postoperatively, both of which were healed after dressing change. There was no other perioperative complication. Postoperatively, the lower limbs were equal in length; the affected limb had no deformities such as shortness and rotation.

#### Typical Case

**Fig. 4:**
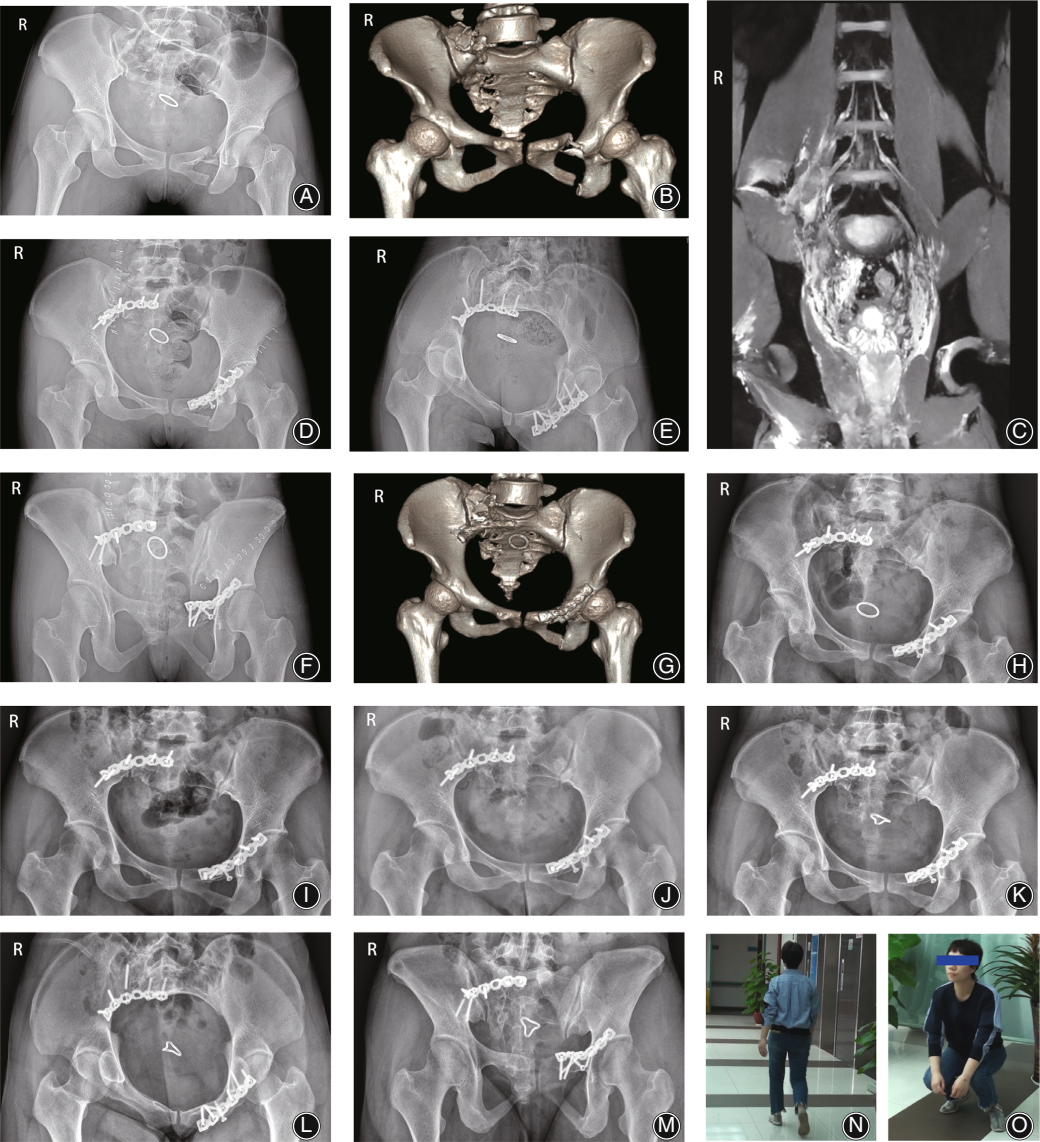
(A) Preoperative AP view; (B) Preoperative 3‐D reconstruction of CT data; (C) Preoperative MRI; (D–F) Postoperative AP, inlet and outlet views; (G) Postoperative 3‐D reconstruction of CT data; (H) Postoperative AP view at 3‐month follow‐up; (I) Postoperative AP view at 2‐year follow‐up; (J) Postoperative AP view at 4‐year follow‐up; (K–M) Postoperative AP, inlet, and outlet views at 8‐year follow‐up; (N–O) The patient could walk, run, and jump normally without complaints of sacroiliac pain at 8‐year follow‐up

A 30‐year‐old female, who was injured in a car accident and sustained pelvic fracture (Young–Burgess classification: *VS*; Tile classification: C1.3) combined with right sacral plexus injury (physical examination, CT, and MRI all suggest the injury of the right sacral plexus), was treated *via* the lateral‐rectus approach (Fig. 4).

## Discussion

In our study, the LRA can well expose the medial hemipelvic ring, which could provide sufficient surgical field for surgical decompression and neurolysis of LSP, satisfactory reduction and fixation. All in all, LRA was a safe and feasible surgical approach for treating the *VS* pelvic fracture combined with LSP injury.

### Feasibility of Using LRA on VS Pelvic Fractures with LSP Injury

Generally, to expose LSP, the SI joint and profundus tissue must be exposed fully, which is highly risky. Usually, the ilioinguinal and modified Stoppa approaches are frequently found anterior surgical approaches for *VS* pelvic fractures. However, the surgical field of either the ilioinguinal approach or modified Stoppa are not large enough to expose both the SI joint and LSP. Therefore, this study was designed, and our results suggested‐that LRA can expose and release the compressed lumbosacral plexus nerve and stretch it safely, and can be conducted simultaneously with ORIF.

LSP injury has an incidence as high as 50% in *VS* pelvic fracture, but controversies still exist in treating *VS* pelvic fracture with LSP injury. The recovery rate of nerve injury has been reported to be 27.3%–92.3% without surgical intervention.^10^ However, Sabiston *et al*.^11^ reported that surgical treatment of LSP injury should be done when conservative treatment fails. In addition, some investigators^12^, ^13^ have reported that early surgery can relieve the compression of the fracture fragments on the nerves and prevent the nerve roots from being continuously pulled. Consistent with these studies, our data exhibited a promising recovery from early surgical treatment from pelvic fractures with LSP injury.

### Safety and Efficacy of Using LRA on VS Pelvic Fractures with LSP Injury

LRA has been applied for treating complicated acetabular fractures, acetabular fractures in the aged, and toddlers' unstable pelvic fractures effectively,^7^, ^14^, ^15^ especially for the dislocated SI joint. Inspired by our recent publications, we used this method for exposing and decompressing LSP injuries; our results indicated the safety and feasibility of using LRA on *VS* pelvic fractures with LSP injury.

Although the operative approach was simple and convenient (mean operating time: 155.2 ± 32.1 min), the operative blood loss was high (mean, 1021.4 ± 363.4 mL). Hence, nerve exposure and release should be performed after diagnosing LSP injury through physical examination, CT, MRI, and operated by senior, experienced surgeons.

### Limitations

The study design had several limitations. First, the study was retrospective, and a control group was absent. Due to the low incidence of *VS* pelvic fracture combined with LSP injury, there were not enough patients to set up the control group. Second, the sample was too small to conclude that one has a convincing perspective of an LRA in an LSP injury. Therefore, in future studies, a larger number of patients will be observed and a longer follow‐up will be incorporated.

## Conclusion

In this study, the LRA could well separate the medial hemipelvic ring, from the sacroiliac joint to the pubic symphysis. The operation can be performed under direct vision to release the lumbosacral trunk being compressed and stretched, achieve a satisfactory reduction, and effectively fix it with a trans‐sacroiliac plate or sacroiliac screw. LRA was a safe and feasible surgical approach for treating the *VS* pelvic fracture combined with LSP injury. Meanwhile, in order to make our view more convincing, future research should collect more cases of *VS* pelvic fracture combined with LSP injury, and include those undergoing conservative treatment as the control group. In a word, the results of this study at this stage have certain guiding significance for clinical practice.

### Authorship Declaration

All authors listed meet the authorship criteria according to the latest guidelines of the International Committee of Medical Journal Editors. Xiaorui Zhan, Kangshuai Xu and Qiubao Zheng contributed equally to this manuscript.
